# Intervention–analog association between traditional Chinese medicine syndrome load and lipid/C-reactive protein biomarkers: a single-center retrospective cohort study using target trial emulation

**DOI:** 10.3389/fmed.2026.1789294

**Published:** 2026-07-06

**Authors:** Yu-Xuan Guo, Chao Han, Xin Xiang, Sheng Wang, Li Yang, Shuang-Jian Li

**Affiliations:** 1 Center Laboratory Medicine, Hospital of Traditional Chinese Medicine Affiliated to Xinjiang Medical University, Urumqi City, Xinjiang Uygur Autonomous Region, China; 2 Department of Breast Surgery, The Affiliated Cancer Hospital of Xinjiang Medical University, Xinjiang Key Laboratory of Oncology, Urumqi, Xinjiang, China

**Keywords:** confirmatory factor analysis, double machine learning, inflammation, inverse probability weighting, lipid metabolism, syndrome differentiation, target trial emulation, traditional Chinese medicine

## Abstract

**Background:**

Traditional Chinese Medicine (TCM) syndrome differentiation is central to individualized diagnosis, but its relationship with objective biochemical markers remains insufficiently characterized within explicit frameworks for intervention–analog causal inference.

**Objectives:**

This study aimed to construct a syndrome load index using confirmatory factor analysis (CFA) and item response theory (IRT), assess measurement invariance, and estimate adjusted intervention–analog associations with lipid and C-reactive protein (CRP) biomarkers.

**Methods:**

This single-center retrospective cohort study included 1,472 adults with structured TCM assessments and repeated biochemical measurements over 90 days. Syndrome load was modeled as a continuous latent variable using CFA/IRT. A Gaussian generalized propensity score with stabilized inverse probability of treatment weighting, combined with double machine learning cross-fitting, estimated associations with low-density lipoprotein-cholesterol (LDL-C), triglycerides (TG), and CRP z-scores, as well as corresponding binary abnormality outcomes. Quartiles were used only for descriptive diagnostics. Negative controls, baseline laboratory sensitivity analyses, weight diagnostics, and E-value analyses assessed robustness.

**Results:**

CFA/IRT models demonstrated good fit (CFI = 0.96, RMSEA = 0.042) and supported metric/scalar invariance across both sex and age groups. The emulated contrast represented a 1-standard deviation (SD) difference in baseline syndrome load as an analytic intervention analog, rather than a literal randomization of a latent construct. After weighting, all absolute standardized mean differences (SMDs) were <0.10, the effective sample size was 1,289, and no material positivity violations were observed. A 1-SD higher syndrome load was associated with higher TG z-scores (+0.18; 95%CI: 0.12–0.24; approximately +0.20 mmol/L), LDL-C z-scores (+0.11; 95%CI: 0.05–0.18; approximately +0.10 mmol/L), and CRP z-scores (+0.16; 95%CI: 0.09–0.23; approximately +0.31 mg/L), corresponding to 3–5% absolute risk increases in biomarker abnormalities. Baseline laboratory sensitivity analyses yielded attenuated but directionally consistent estimates (TG + 0.16, LDL-C + 0.09, CRP + 0.13 to +0.14 z-score units). Associations were stronger among participants aged ≥65 years, those with diabetes, or those with body mass index (BMI) ≥ 28. Negative controls were null, and E-values ranged from 1.54 to 1.92.

**Conclusion:**

Higher syndrome load was consistently associated with dyslipidemia and CRP elevation within an explicit intervention–analog framework. These findings support syndrome objectification and personalized cardiometabolic risk stratification; however, prospective intervention studies are required before concluding that the latent syndrome construct itself is directly modifiable.

## 1 Introduction

Traditional Chinese Medicine (TCM) syndrome differentiation (辨证论治, bian zheng lun zhi) represents a diagnostic paradigm that integrates multi-dimensional clinical manifestations into coherent patterns reflecting underlying pathophysiological imbalances (1). Despite more than three millennia of empirical refinement, syndrome concepts face persistent challenges in modern evidence-based practice, particularly regarding their objectification through measurable biomarkers and their interpretation in temporally ordered clinical outcome frameworks (2, 3).

Current research on TCM syndromes predominantly uses descriptive approaches—including cross-sectional surveys documenting syndrome–biomarker co-occurrence (4), cluster analyses identifying symptom groupings (5), or correlation studies suggesting associations between specific syndromes and laboratory abnormalities (6). While informative, these methods cannot distinguish whether syndrome indicators precede metabolic dysregulation, whether both phenomena share common upstream determinants, or whether reverse causation operates. This evidential gap limits clinical translation because syndrome assessment requires transparent effect size estimation, explicit time-zero definition, and careful separation of measurement validity from causal identification before it can inform risk stratification or intervention planning (7).

The theoretical plausibility of syndrome–biomarker links merits consideration. Emerging mechanistic studies suggest syndrome manifestations may map onto inflammatory cascades, insulin resistance pathways, and lipid metabolic disruptions (8, 9). For instance, dampness–heat syndrome correlates with elevated pro-inflammatory cytokines (10), while qi-deficiency patterns have been associated with metabolic dysfunction (11). However, these mechanistic hypotheses require validation through rigorous real-world causal inference designs that address confounding, selection bias, time-zero ambiguity, and unmeasured factors inherent in observational TCM data (12).

Methodologically, several innovations can strengthen syndrome–biomarker research while maintaining interpretation appropriately cautious. Target trial emulation (TTE) provides a principled framework for designing observational analyses by explicitly specifying eligibility criteria, exposure definitions, follow-up periods, time zero, and analytic plans (13–15). Since syndrome load is a reflective latent construct rather than a directly randomized treatment, the present estimand is conceptualized as an intervention analog: The expected biomarker contrast associated with a 1-SD difference in baseline syndrome load could arise from syndrome-targeted clinical management, symptom regulation, and adherence support, without implying that the latent score itself can be selectively manipulated. Measurement invariance testing using CFA and IRT supports meaningful interpretation of latent scores and subgroup comparisons, although it does not by itself establish exchangeability or confounding control (16, 17). Double machine learning (DML) combined with Inverse probability of treatment weighting‌ (IPTW) offers doubly robust estimators by leveraging flexible machine learning while maintaining valid inference under stated assumptions (18, 19). Finally, negative control exposures/outcomes and E-values provide complementary sensitivity analyses for unmeasured confounding, a ubiquitous threat in observational TCM research (20–22).

Clinically, reproducible syndrome–biomarker associations could support TCM practice by providing an interpretable bridge between syndrome differentiation and conventional cardiometabolic risk assessment. A validated syndrome load index could complement conventional risk scores (e.g., Framingham and SCORE2), identify high-risk individuals for closer monitoring and guideline-aligned short-term reassessment, and generate testable hypotheses for syndrome-targeted intervention trials (23–25). Furthermore, objective syndrome quantification may facilitate the integration of TCM diagnostics into electronic health records and enable large-scale epidemiological investigations (26).

The present study addresses these gaps by constructing and validating a syndrome load index through latent variable modeling and measurement invariance testing, explicitly specifying a target-trial-inspired intervention–analog contrast, implementing a continuous generalized propensity score with stabilized IPTW combined with DML to estimate average and heterogeneous associations, and conducting rigorous sensitivity analyses to assess robustness to unmeasured confounding.

## 2 Methods

### 2.1 Study design and data sources

This single-center retrospective cohort study applied target trial emulation principles to estimate adjusted intervention–analog associations between TCM syndrome load, assessed using structured four-diagnostic methods, and lipid/CRP biomarkers over a 90-day follow-up period. Data were extracted from the hospital information system and electronic medical records, encompassing demographics, TCM diagnostic assessments, laboratory results, medications, and clinical complications, with linkage ensured via unique patient identifiers. The inclusion criteria included individuals aged ≥18 years; those who had completed a baseline four-diagnostic assessment including tongue, pulse, and symptom domains; those with availability of LDL-C, TG, and CRP measurements at baseline and within the 90 ± 14-day follow-up window; and those with a clinical diagnosis or management goals related to dyslipidemia or metabolic risk. The exclusion criteria included individuals with acute severe infections or hospitalizations potentially altering CRP acutely, active cancer chemotherapy, pregnancy, or missing critical covariates not amenable to multiple imputation. This study received institutional review board approval with a waiver of informed consent for de-identified secondary data analysis.

#### 2.1.1 Explicit target trial emulation specification

The target trial specification was used as a design discipline rather than as a claim that syndrome load itself could be directly randomized. Eligible adults with complete baseline syndrome assessment and baseline biomarker profiling entered the emulation at the date of the index TCM evaluation (time zero). The exposure strategy was defined as a clinical syndrome-reduction strategy expected to reduce observable syndrome manifestations and thereby shift the standardized syndrome load distribution by 1 SD; the 1-SD unit was an analytic scaling device rather than a clinical procedure. The comparator was the observed higher syndrome load state. Assignment in the emulation was modeled through a continuous generalized propensity score conditional on prespecified pre-time-zero confounders. Index-visit decisions initiated after the baseline assessment, including diet counseling, herbal prescribing, or lipid-lowering treatment intensification, were treated as post-baseline co-interventions and were not conditioned upon as baseline confounders to avoid immortal time and mediator bias. Baseline medication use documented before time zero was included in the confounder set. The follow-up period was extended to 90 days to capture short-term CRP fluctuation and early lipid response within the 4- to 12-week reassessment window used in lipid management while minimizing attrition and treatment crossover in routine care (23). Detailed outcome definitions are provided in Section 2.2.2. The estimand was interpreted as the adjusted intervention–analog association associated with a 1-SD shift in syndrome load, and the analytic plan prespecified stabilized IPTW, cross-fitted DML, subgroup-specific Conditional average treatment effect (CATE) estimation, negative controls, and E-value-based sensitivity analyses.

### 2.2 Variable definitions and latent variable modeling

#### 2.2.1 Exposure: syndrome load index via CFA and IRT

Syndrome load represented the primary exposure, conceptualized as a continuous latent variable reflecting overall syndrome load. Structured four-diagnostic items (tongue characteristics, pulse qualities, and symptom clusters) were pre-assigned to theoretically grounded domains based on expert consensus: dampness phlegm (湿痰), dampness–heat (湿热), qi-stagnation (气滞), blood stasis (血瘀), spleen deficiency (脾虚), and liver qi depression (肝郁). A confirmatory factor analysis evaluated model fit and tested measurement invariance across sex and age groups following established protocols. Fit indices included the comparative fit index (CFI ≥ 0.95), Tucker–Lewis index (TLI ≥ 0.95), root mean square error of approximation (RMSEA ≤0.06), and standardized root mean square residual (SRMR ≤0.08). Multi-group invariance testing proceeded hierarchically: configural invariance (same factor structure), metric invariance (equal factor loadings), and scalar invariance (equal item intercepts). For items exhibiting ordinal response patterns, graded response models (GRM) or two-parameter logistic IRT models estimated person-level latent trait scores (*θ*). The final latent scores underwent linear transformation to standardized z-scores (mean = 0, SD = 1), defined as the “syndrome load index.”

#### 2.2.2 Outcomes and follow-up windows

Primary continuous outcomes included z-scores for LDL-C, TG, and CRP, standardized using center-specific contemporary reference population means and standard deviations (LDL-C SD 0.88 mmol/L, TG SD 1.09 mmol/L, and CRP SD 1.95 mg/L). Primary binary outcomes defined abnormalities using clinical thresholds: LDL-C ≥ 3.4 mmol/L (130 mg/dL), TG ≥ 1.7 mmol/L (150 mg/dL), and CRP ≥ 3.0 mg/L. Secondary outcomes comprised the first hyperlipidemia-related treatment intensification after 90 days and persistently elevated CRP (baseline and follow-up CRP ≥ 3.0 mg/L). The 90-day window was selected *a priori* to align with routine outpatient reassessment, capture clinically interpretable short-term CRP and lipid changes, and remain close to lipid-treatment reassessment intervals commonly used after therapy or lifestyle changes (23).

#### 2.2.3 Covariates and potential confounders

Baseline covariates captured established determinants of both syndrome patterns and biomarker abnormalities: (1) demographic data (age, sex, and body mass index [BMI]); (2) comorbidities (hypertension, type 2 diabetes mellitus, chronic kidney disease stage via estimated glomerular filtration rate [eGFR], smoking status, and alcohol use); (3) medications documented before time zero (statins, fibrates, aspirin, metformin, and antihypertensive agents); (4) baseline laboratory values measured before or at the index assessment (total cholesterol, LDL-C, TG, HDL-C, fasting glucose, alanine aminotransferase, aspartate aminotransferase, creatinine, and CRP for balance transparency); and (5) lifestyle factors assessed via validated questionnaires. Baseline laboratory variables were treated as time-zero severity proxies rather than post-baseline outcomes; since baseline CRP may partly reflect early inflammatory expression of syndrome manifestations, CRP estimates were interpreted as adjusted intervention–analog associations rather than controlled direct effects. No post-baseline biomarker values or treatment changes were entered as confounders. Variables underwent outlier detection and consistency checks pre-analysis.

### 2.3 Data preprocessing and missing data

#### 2.3.1 Missing data assessment and multiple imputation

Missing data patterns were characterized using Little’s test for missing completely at random (MCAR). Given the plausible missing-at-random (MAR) mechanism, we implemented multivariate imputation by chained equations (MICE) with 20 imputed datasets (m = 20). Continuous variables used predictive mean matching; binary/categorical variables used a logistic or polytomous regression analysis; ordinal syndrome items used ordered logistic regression analysis within the imputation chains. Each imputed dataset underwent independent latent variable estimation and causal analysis, with results pooled via Rubin’s rules.

### 2.4 Propensity score modeling and inverse probability weights

#### 2.4.1 Stabilized IPTW and covariate balance

Syndrome load was modeled continuously in the primary propensity analysis using a Gaussian generalized propensity score; quartiles were used only for descriptive balance diagnostics and visualization. The conditional density of syndrome load relative to baseline covariates was estimated using super learner ensembles incorporating generalized linear models, generalized additive models, and random forests. Stabilized weights were calculated as the marginal density of the observed syndrome load divided by the conditional density, preventing extreme weight inflation. Extreme weights underwent sensitivity analyses through percentile-based truncation (1st/99th percentiles and neighboring alternatives) and fixed upper-bound diagnostics. Covariate balance was assessed using standardized mean differences (SMDs), with |SMD| < 0.1 indicating adequate balance. For residual imbalances, covariate balancing propensity scores (CBPS) were used as a robustness check.

### 2.5 Double machine learning framework

#### 2.5.1 Cross-fitting and orthogonalization

Double machine learning implemented Neyman orthogonalization to achieve bias reduction and valid inference when nuisance parameters were estimated via flexible machine learning. The samples were randomly partitioned into K = 5 folds for cross-fitting. In each fold, nuisance functions—the conditional expectations of outcome given covariates (E[Y|X]) and treatment given covariates (E[D|X])—were estimated on the training folds using super learner ensembles. The SuperLearner ensemble combined least absolute shrinkage and selection operator (LASSO) regression, random forests, extreme gradient boosting, and elastic net. Residualized outcomes and residualized treatments from the validation fold were then regressed to obtain approximately unbiased, root-n consistent ATE estimates. For continuous outcomes (z-scores), we applied linear orthogonal moments; for binary outcomes, partially linear logistic regression analysis with orthogonal scores.

#### 2.5.2 Augmented IPTW and heterogeneous associations

Within the DML framework, we combined orthogonalization with IPTW to construct augmented inverse probability weighting (AIPW) estimators—doubly robust because they remain consistent if either the outcome model or the propensity model is correctly specified. To explore treatment effect heterogeneity (CATE), we used T-learner and S-learner meta-learners. SHapley Additive exPlanations (SHAP) values and causal forests identified key effect modifiers. Prespecified subgroups for CATE estimation included (1) sex, (2) age (<65 vs. ≥65 years), (3) diabetes status, (4) statin use at baseline, and (5) BMI (<25, 25–28, ≥28 kg/m^2^).

### 2.6 Negative controls and unmeasured confounding assessment

#### 2.6.1 Negative control exposures and outcomes

To detect residual confounding or model misspecification, we implemented prespecified negative controls selected using three criteria: absence of a plausible 90-day biological pathway to lipid/CRP regulation, absence of a substantive overlap with the core syndrome domains used to define the exposure, and shared dependence on examination workflow or documentation conditions sufficient to probe data capture bias (20). The negative control exposure was a procedural pseudo-score composed of rank-normalized examination-order indicators, tongue image illumination artifact residuals after routine white balance correction, and non-dominant wrist pulse recording order. These components were standardized and averaged because they reflect data capture conditions rather than cardiometabolic biology while still sharing clinic flow and documentation processes with syndrome assessment. The negative control outcome was the incident documentation of a simple renal cortical cyst notation on routine ultrasonography, a benign imaging finding not expected to be altered by short-term syndrome load variation. Null negative control results were interpreted as evidence against gross workflow-related bias, not as proof that all unmeasured confounding had been eliminated.

#### 2.6.2 E-value calculation

For each primary association estimate, we calculated E-values—the minimum strength of association on the risk ratio scale that an unmeasured confounder would require with both syndrome load and outcomes to fully explain the observed associations, conditional on measured covariates (21, 22). For binary outcomes, E-values were computed directly from risk ratio approximations derived from the estimated risk differences and baseline risks. For continuous outcomes, standardized mean differences were translated to approximate risk ratios using exp.(0.91 x SMD) before E-value calculation, and both point estimate and confidence-limit E-values were reported. These values were contextualized by comparing them to the strength of measured confounders in the weighted cohort.

#### 2.6.3 Baseline laboratory sensitivity analyses

To address potential overconditioning by baseline laboratory values, particularly baseline CRP, we performed additional sensitivity analyses using two reduced adjustment specifications. First, the CRP-specific GPS/DML nuisance models were repeated after omitting baseline CRP while retaining demographic factors, lifestyle factors, comorbidities, pre-time-zero medication use, and non-CRP laboratory severity markers. Second, a reduced laboratory model excluded baseline inflammatory and metabolic biomarkers from the propensity score while retaining core clinical confounders. These analyses were interpreted as robustness checks for direction and approximate magnitude rather than as separate primary estimands because baseline biomarkers may represent both pre-time-zero severity and early expression of syndrome manifestations.

### 2.7 Statistical inference and multiple comparison adjustment

#### 2.7.1 Confidence intervals and error rate control

Average intervention–analog association estimates and CATE-style estimates were reported with 95% confidence intervals derived via cross-fit bootstrapping (1,000 iterations) or asymptotic approximations based on influence functions. For multiple outcome testing (LDL-C, TG, CRP z-scores, and binary outcomes), we applied Benjamini–Hochberg false discovery rate (FDR) control at *α* = 0.05. Two-sided *p*-values of <0.05 (after FDR adjustment) were considered statistically significant.

### 2.8 Precision and statistical power

#### 2.8.1 Detectable effect assessment

Given the observational design with a fixed sample size (*N* = 1,472 enrolled, *N* = 1,356 with complete 90-day follow-up), precision was assessed by estimating detectable effect sizes under the achieved effective sample size after weighting. For continuous outcomes, the analysis was powered to detect clinically meaningful differences of approximately 0.15 SD in biomarker z-scores per 1-SD syndrome load increase. For binary outcomes, calculations assumed a baseline prevalence of 35–45% for LDL-C/TG abnormalities and evaluated detectable absolute risk differences (ARD). Calculations incorporated design effects from weighting.

### 2.9 Software and reproducibility

Analyses were conducted in R version 4.3.1 using the following packages: *lavaan* for CFA, *mirt* for IRT, *mice* for imputation, *WeightIt* and *twang* for propensity scores, *DoubleML* for DML, *EValue* for sensitivity analyses, and *tidyverse* for data manipulation. The analysis code, curated metadata dictionary, and a synthetic dataset with the same variable structure will be deposited in a public repository after publication. Real patient-level data are available from the corresponding author on reasonable request, subject to institutional approval and privacy safeguards.

## 3 Results

### 3.1 Cohort assembly and baseline characteristics

**Figure 1:**
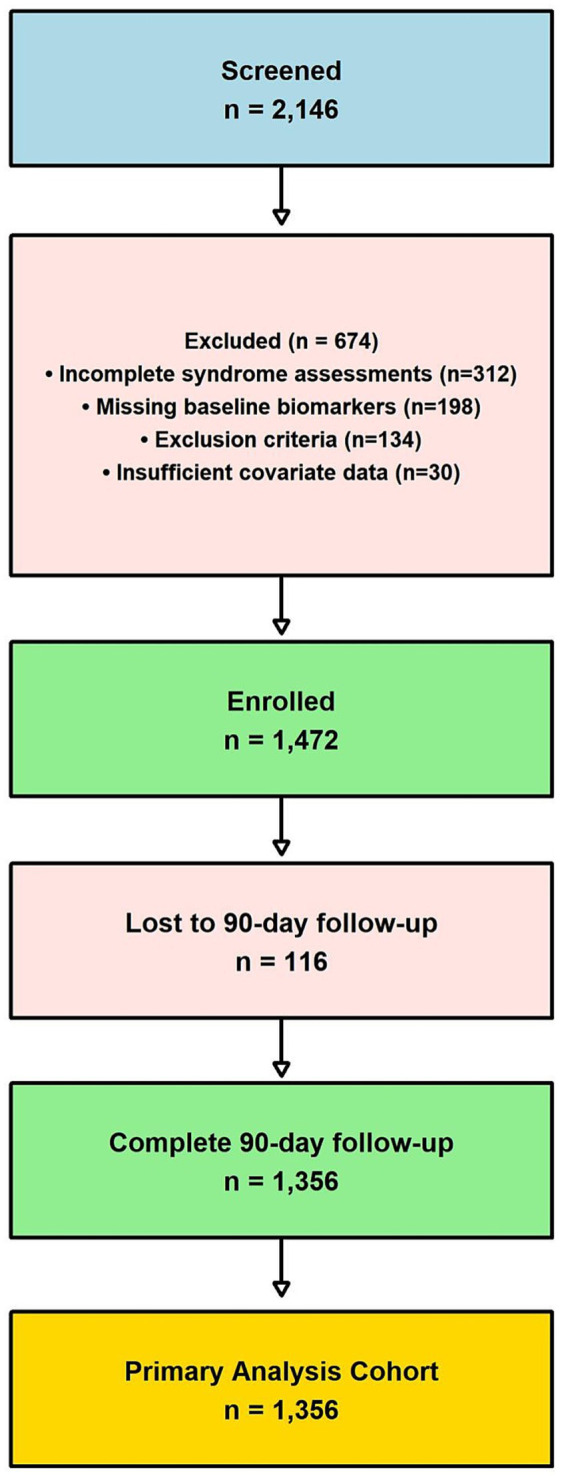
CONSORT-style flowchart of participant selection. Boxes indicate screening, exclusions with reasons, enrollment, and 90-day follow-up completion.

Figure 1 shows participant flow. Among 2,146 individuals screened, 674 were excluded for incomplete syndrome assessments (*n* = 312), missing baseline biomarkers (*n* = 198), exclusion criteria (*n* = 134), or insufficient covariate data (*n* = 30), yielding 1,472 enrolled participants. At the 90-day follow-up, 116 lacked repeat biochemical testing, resulting in 1,356 individuals for primary analyses. Table 1 summarizes baseline characteristics. The mean age was 58.3 years (SD = 11.7), with 52.8% being female. The baseline mean LDL-C was 3.2 mmol/L (SD = 0.9), TG was 1.8 mmol/L (SD = 1.1), and CRP was 2.4 mg/L (interquartile range [IQR]: 1.1–4.8). Syndrome load index exhibited approximately normal distribution (mean = 0.02, SD = 0.98, skewness = 0.14). The prevalence of baseline lipid abnormalities was as follows: LDL-C ≥ 3.4 mmol/L (42.1%), TG ≥ 1.7 mmol/L (47.6%), and CRP ≥ 3 mg/L (38.3%).

**Table 1:** Baseline characteristics of study participants (*N* = 1,472).

Characteristic	Value
Age, years (mean ± SD)	58.3 ± 11.7
Female sex, *n* (%)	777 (52.8)
Body mass index, kg/m^2^ (mean ± SD)	25.1 ± 3.6
Hypertension, *n* (%)	612 (41.6)
Type 2 diabetes mellitus, *n* (%)	384 (26.1)
Current smoking, *n* (%)	294 (20.0)
Alcohol use, *n* (%)	221 (15.0)
Statin use at baseline, *n* (%)	556 (37.8)
Fibrate use at baseline, *n* (%)	118 (8.0)
Baseline laboratory values	
LDL-cholesterol, mmol/L (mean ± SD)	3.2 ± 0.9
Triglycerides, mmol/L (mean ± SD)	1.8 ± 1.1
HDL-cholesterol, mmol/L (mean ± SD)	1.3 ± 0.3
Total cholesterol, mmol/L (mean ± SD)	5.1 ± 1.1
C-reactive protein, mg/L (median [IQR])	2.4 [1.1–4.8]
Fasting glucose, mmol/L (mean ± SD)	5.8 ± 1.4
eGFR, mL/min/1.73m^2^ (mean ± SD)	84.2 ± 18.6
Binary abnormality prevalence	
LDL-C ≥ 3.4 mmol/L, *n* (%)	620 (42.1)
TG ≥ 1.7 mmol/L, *n* (%)	701 (47.6)
CRP ≥ 3.0 mg/L, *n* (%)	564 (38.3)
Syndrome Load Index	
Mean ± SD	0.02 ± 0.98
Median [IQR]	0.01 [−0.64 to 0.68]

SD, standard deviation; IQR, interquartile range; LDL-C, low-density lipoprotein cholesterol; TG, triglycerides; HDL-C, high-density lipoprotein cholesterol; CRP, C-reactive protein; eGFR, estimated glomerular filtration rate.

### 3.2 Latent variable modeling and measurement invariance

CFA models demonstrated excellent fit across all indices (Table 2). The baseline model achieved CFI = 0.962, TLI = 0.951, RMSEA = 0.042 (90%CI: 0.037–0.047), and SRMR = 0.038. Multi-group invariance testing across sex (male vs. female) and age strata (<65 vs. ≥65 years) supported scalar invariance: metric invariance models showed minimal CFI decrements (ΔCFI = 0.004–0.007, all <0.01 threshold) and comparable fit indices. Scalar invariance models exhibited ΔCFI ≤0.007, satisfying criteria for significant mean comparisons. Factor loadings ranged from *λ* = 0.52 to λ = 0.84 across syndrome domains, with tongue and pulse items contributing strongly to latent factor identification. IRT analyses for polytomous items confirmed adequate item discrimination (a-parameters 0.9–2.1) and appropriate threshold spacing.

**Table 2:** Latent variable model fit and measurement invariance testing.

Model	CFI	TLI	RMSEA	SRMR	ΔCFI vs. Baseline
Baseline configural model	0.962	0.951	0.042	0.038	—
Sex invariance					
Configural (sex groups)	0.960	0.949	0.043	0.040	−0.002
Metric (equal loadings)	0.958	0.948	0.044	0.040	−0.004
Scalar (equal intercepts)	0.956	0.946	0.045	0.041	−0.006
Age invariance (<65 vs. ≥65 years)					
Configural (age groups)	0.961	0.950	0.043	0.039	−0.001
Metric (equal loadings)	0.959	0.949	0.044	0.040	−0.003
Scalar (equal intercepts)	0.955	0.945	0.046	0.042	−0.007

CFI, comparative fit index; TLI, Tucker-Lewis index; RMSEA, root mean square error of approximation; SRMR, standardized root mean square residual; ΔCFI, change in CFI relative to baseline model. Note: Metric and scalar invariance models satisfied ΔCFI<0.01 criterion, supporting measurement equivalence across groups.

**Figure 2:**
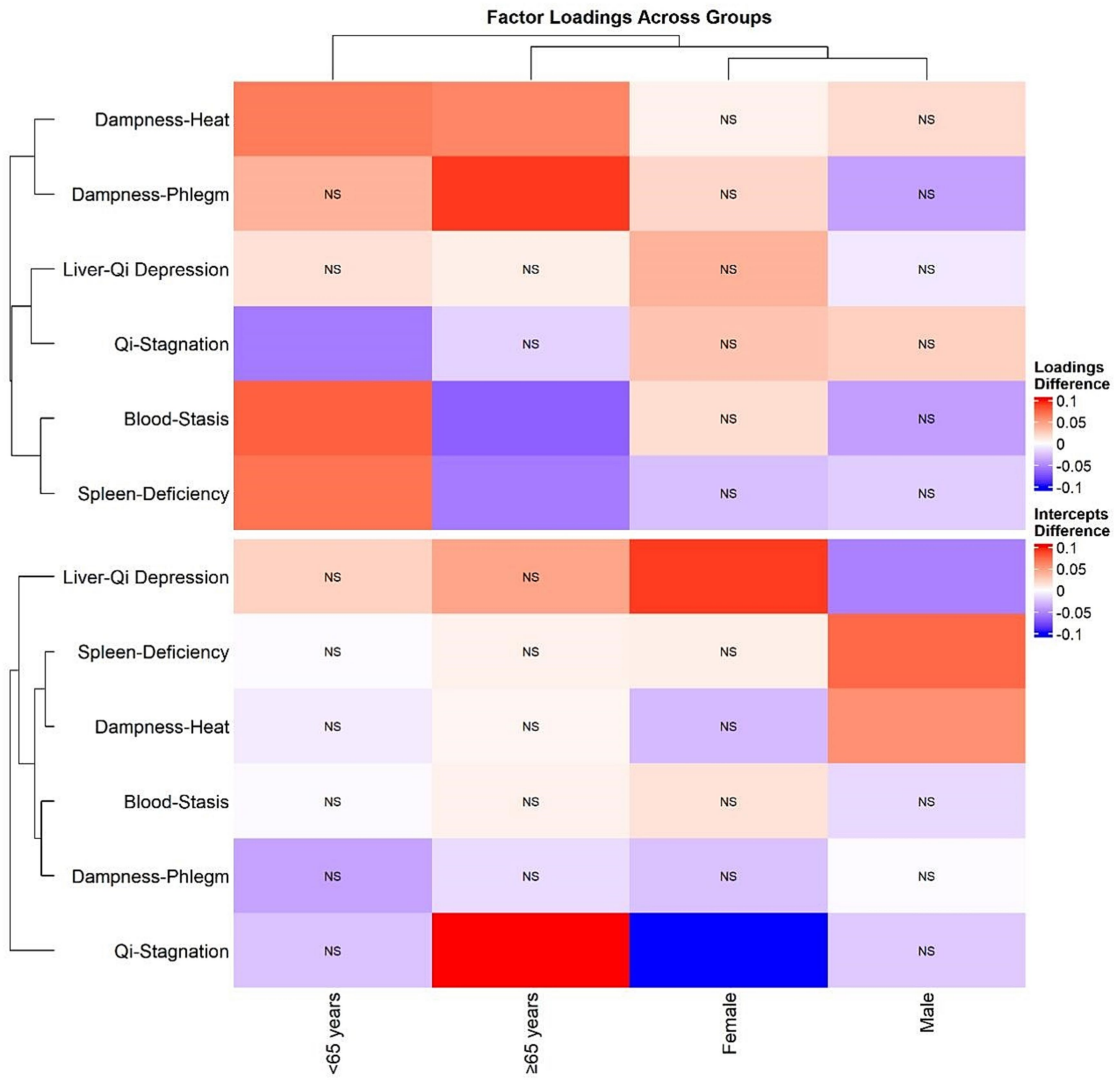
Measurement invariance heatmap.

Figure 2 presents a heatmap visualization of measurement invariance testing results across demographic subgroups, demonstrating consistent factor structure and item performance across sex and age strata.

### 3.3 Propensity score balance

Before IPTW, several covariates exhibited imbalances across syndrome load strata (Table 3). Notably, individuals with higher syndrome load were more likely to have diabetes (SMD = 0.24), higher BMI (SMD = 0.22), more frequent statin use (SMD = 0.21), and higher baseline CRP, underscoring the need to interpret inflammatory outcomes with attention to time-zero severity. After applying stabilized GPS-based IPTW with 1%/99% truncation, all balance-diagnostic covariates achieved absolute SMDs <0.10, the weighted effective sample size was 1,289, and the mean stabilized weight was 1.02 (SD: 0.18; 1%/99%: 0.61–1.74; maximum truncated weight: 2.94), indicating adequate measured overlap without extreme positivity violations. Supplementary Figure S1 shows the pre- versus post-weighting Love plot, and Supplementary Figure S2 shows the stabilized weight distribution.

**Table 3:** Covariate balance and weight diagnostics before and after continuous generalized propensity score weighting.

Covariate	SMD before IPTW	SMD after IPTW	Weighted variance ratio
Age	0.18	0.04	1.01
Female sex	0.07	0.02	0.98
Body mass index	0.22	0.06	1.03
Hypertension	0.14	0.05	1.01
Type 2 diabetes	0.24	0.07	1.04
Current smoking	0.11	0.03	0.99
Statin use	0.21	0.05	1.02
Baseline LDL-C	0.16	0.04	1.05
Baseline TG	0.19	0.05	1.04
Baseline HDL-C	0.19	0.05	0.97
Baseline CRP	0.20	0.06	1.03
Fasting glucose	0.17	0.04	1.02
eGFR	0.13	0.03	0.96
ALT	0.15	0.04	1.01
Effective sample size	–	1,289	–
Mean stabilized weight (SD)	–	1.02 (0.18)	–
Weight percentile range (1st-99th)	–	0.61–1.74	–

SMD, standardized mean difference; IPTW, inverse probability of treatment weighting; LDL-C, low-density lipoprotein cholesterol; TG, triglycerides; HDL-C, high-density lipoprotein cholesterol; CRP, C-reactive protein; eGFR, estimated glomerular filtration rate; ALT, alanine aminotransferase. Absolute SMD<0.1 and variance ratios near 1.0 indicate adequate balance; the primary exposure was modeled continuously using a Gaussian generalized propensity score, and quartiles were used only for diagnostics. Baseline CRP is reported for balance transparency and is interpreted as a time-zero severity marker rather than as evidence that CRP-outcome estimates are controlled direct effects.

### 3.4 Primary intervention–analog associations

Table 4 presents primary association estimates from the DML + AIPW+GPS-IPTW analyses. Each 1-SD higher syndrome load was associated with significant increases in all primary biomarker z-scores: TG z-score increased by +0.18 (95%CI: 0.12–0.24, *p* < 0.001), corresponding to approximately +0.20 mmol/L; LDL-C z-score increased by +0.11 (95%CI: 0.05–0.18, *p* = 0.001), corresponding to approximately +0.10 mmol/L; and CRP z-score increased by +0.16 (95%CI: 0.09–0.23, p < 0.001), corresponding to approximately +0.31 mg/L. For binary outcomes, syndrome load increments corresponded to absolute risk increases of +4.6% (95%CI: +2.1% to +7.1%) for TG ≥ 1.7 mmol/L, +3.1% (95%CI: +0.8% to +5.5%) for LDL-C ≥ 3.4 mmol/L, and +3.8% (95%CI: +1.4% to +6.2%) for CRP ≥ 3.0 mg/L. All associations remained significant after the Benjamini–Hochberg correction.

**Table 4:** Primary intervention-analogue associations of syndrome load with lipid and CRP outcomes.

Outcome	Effect measure	Estimate	95% CI	Approximate absolute change	*p*-value	FDR-adjusted *p*
Continuous outcomes						
TG z-score	Delta per 1-SD syndrome load	+0.18	0.12 to 0.24	~ + 0.20 mmol/L TG	<0.001	<0.001
LDL-C z-score	Delta per 1-SD syndrome load	+0.11	0.05 to 0.18	~ + 0.10 mmol/L LDL-C	0.001	0.002
CRP z-score	Delta per 1-SD syndrome load	+0.16	0.09 to 0.23	~ + 0.31 mg/L CRP	<0.001	<0.001
Binary abnormality outcomes						
TG ≥ 1.7 mmol/L	Absolute risk difference (%)	+4.6	+2.1 to +7.1	Absolute risk +4.6%	<0.001	<0.001
LDL-C ≥ 3.4 mmol/L	Absolute risk difference (%)	+3.1	+0.8 to +5.5	Absolute risk +3.1%	0.008	0.012
CRP ≥ 3.0 mg/L	Absolute risk difference (%)	+3.8	+1.4 to +6.2	Absolute risk +3.8%	0.003	0.005

TG, triglycerides; LDL-C, low-density lipoprotein cholesterol; CRP, C-reactive protein; CI, confidence interval; FDR, false discovery rate. Estimates were derived from double machine learning with augmented inverse probability weighting using a continuous generalized propensity score. The 1-SD syndrome-load contrast is interpreted as an intervention-analogue association, not as literal randomization of a latent construct. Approximate absolute changes were obtained by multiplying z-score estimates by the center-specific reference standard deviation.

**Figure 3:**
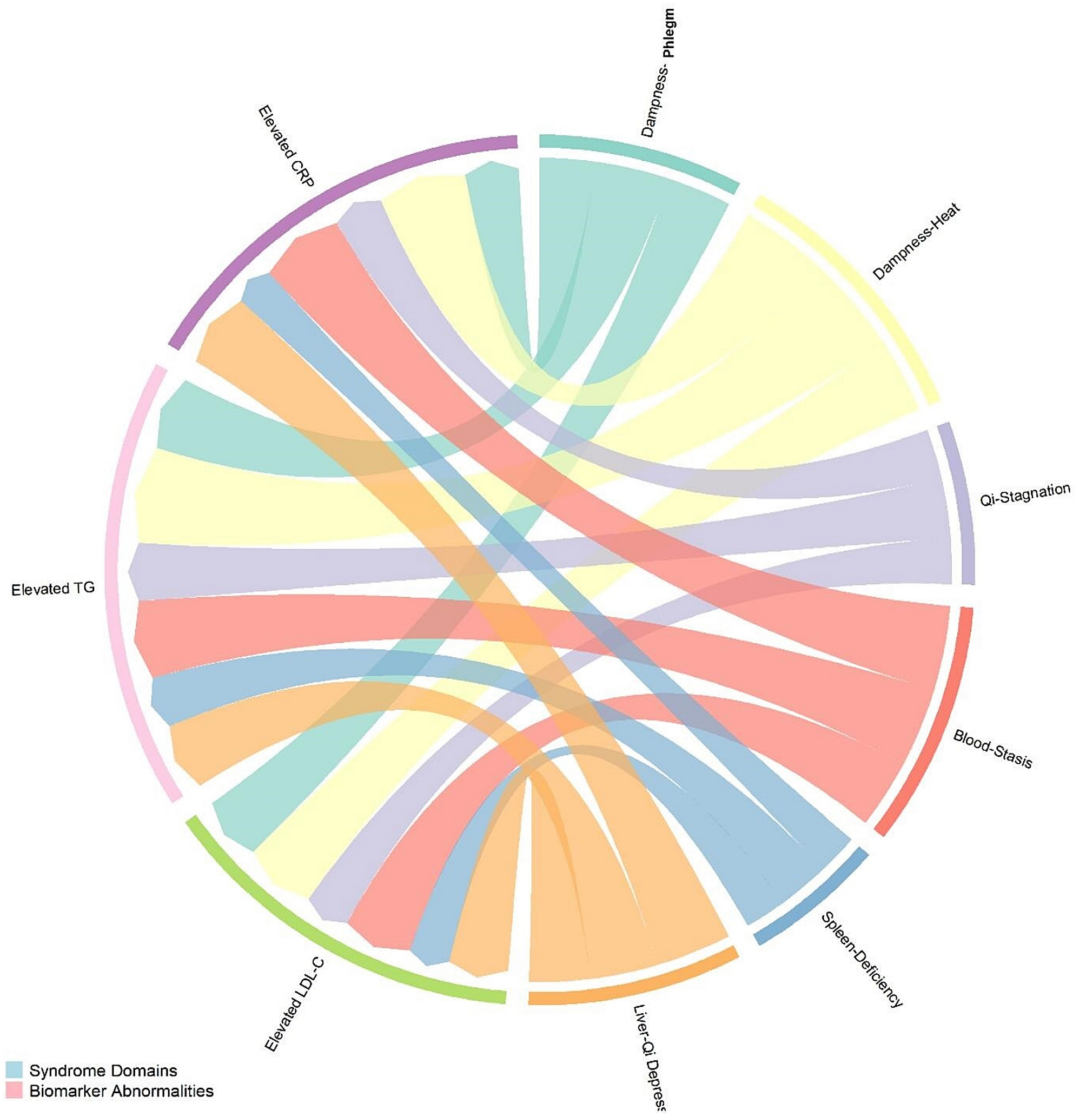
Chord diagram visualizing syndrome domain–biomarker abnormality associations.

**Figure 4:**
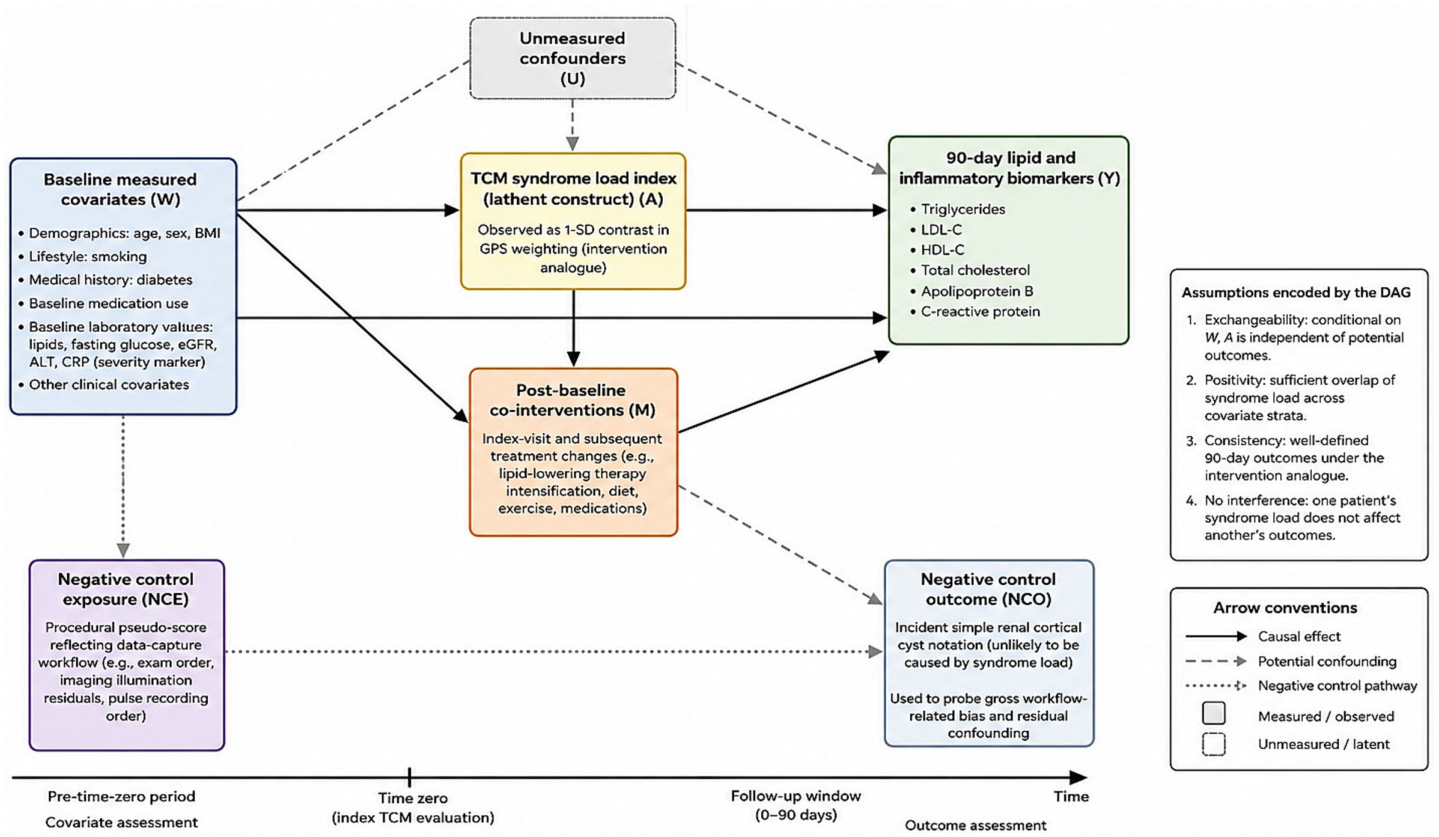
Directed acyclic graph (DAG) for the target-trial-inspired intervention–analog framework. The DAG distinguishes pre-time-zero measured covariates, the latent syndrome load index, post-baseline co-interventions, follow-up lipid/inflammatory biomarkers, unmeasured confounding, and negative controls; syndrome load is explicitly labeled as a latent exposure index rather than a directly randomized treatment.

Figure 3 visualizes the network of associations between specific syndrome domains and biomarker abnormalities using a chord diagram. Figure 4 shows the directed acyclic graph (DAG) underlying the target-trial-inspired design and bias assessment. The DAG labels syndrome load as a latent exposure index rather than a directly randomized treatment, separates pre-time-zero measured covariates from post-baseline co-interventions, and clarifies that GPS-IPTW and DML adjust only for measured pre-time-zero confounding.

### 3.5 Heterogeneous intervention–analog associations

**Figure 5:**
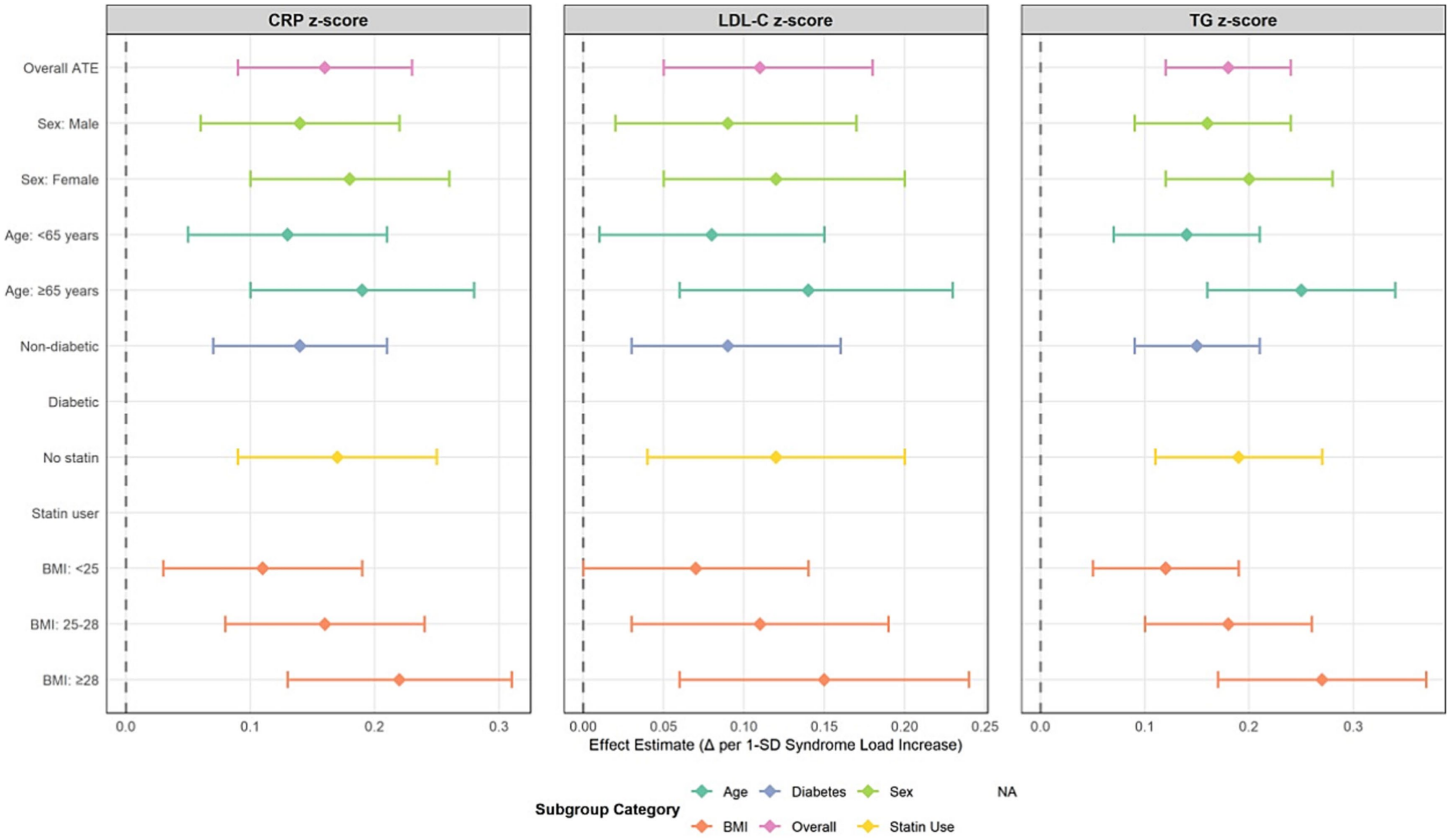
Forest plot of average and subgroup-specific CATE-style intervention–analog association estimates.

CATE-style analyses revealed substantial effect modification (Table 5; Figure 5). Syndrome load associations with biomarker z-scores were consistently stronger among: (1) individuals aged ≥65 years (TG z-score CATE = +0.25, 95%CI: 0.16–0.34 vs. age <65: +0.14, 95%CI: 0.07–0.21; interaction *p* = 0.021); (2) those with diabetes (TG z-score CATE = +0.23, 95%CI: 0.14–0.32 vs. non-diabetic: +0.15, 95%CI: 0.09–0.21; interaction *p* = 0.042); and (3) individuals with BMI ≥ 28 kg/m^2^ (TG z-score CATE = +0.27, 95%CI: 0.17–0.37 vs. BMI < 25: +0.12, 95%CI: 0.05–0.19; interaction *p* = 0.007). Sex-specific differences were modest, with slightly larger associations in women. Statin use did not substantially modify associations, suggesting that syndrome load may capture residual inflammatory or metabolic risk not exhausted by lipid-lowering therapy.

**Table 5:** Conditional average treatment effect-style estimates (CATE) in prespecified subgroups.

Subgroup	TG Z-score CATE	LDL-C Z-score CATE	CRP Z-score CATE	TG abnormality ARD (%)
Sex				
Male	+0.16 (0.09–0.24)	+0.09 (0.02–0.17)	+0.14 (0.06–0.22)	+3.8 (1.2–6.4)
Female	+0.20 (0.12–0.28)	+0.12 (0.05–0.20)	+0.18 (0.10–0.26)	+5.3 (2.6–8.0)
Age				
<65 years	+0.14 (0.07–0.21)	+0.08 (0.01–0.15)	+0.13 (0.05–0.21)	+3.1 (0.8–5.4)
≥65 years	+0.25 (0.16–0.34)	+0.14 (0.06–0.23)	+0.19 (0.10–0.28)	+6.2 (3.4–9.0)
Diabetes status				
Non-diabetic	+0.15 (0.09–0.21)	+0.09 (0.03–0.16)	+0.14 (0.07–0.21)	+3.9 (1.8–6.0)
Diabetic	+0.23 (0.14–0.32)	+0.13 (0.04–0.22)	+0.20 (0.11–0.29)	+5.7 (2.9–8.5)
Statin use				
No statin	+0.19 (0.11–0.27)	+0.12 (0.04–0.20)	+0.17 (0.09–0.25)	+4.8 (2.3–7.3)
Statin user	+0.17 (0.09–0.25)	+0.10 (0.02–0.18)	+0.15 (0.07–0.23)	+4.4 (1.8–7.0)
BMI categories				
<25 kg/m^2^	+0.12 (0.05–0.19)	+0.07 (0.00–0.14)	+0.11 (0.03–0.19)	+2.9 (0.5–5.3)
25–28 kg/m^2^	+0.18 (0.10–0.26)	+0.11 (0.03–0.19)	+0.16 (0.08–0.24)	+4.5 (2.0–7.0)
≥28 kg/m^2^	+0.27 (0.17–0.37)	+0.15 (0.06–0.24)	+0.22 (0.13–0.31)	+6.5 (3.8–9.2)

Values represent model-based subgroup association estimate (95% confidence interval). CATE is retained as the machine-learning terminology for heterogeneity estimation and does not imply literal randomization of the latent syndrome construct. TG, triglycerides; LDL-C, low-density lipoprotein cholesterol; CRP, C-reactive protein; ARD, absolute risk difference; BMI, body mass index.

### 3.6 Sensitivity analyses and robustness

Negative control analyses demonstrated null associations, supporting the absence of gross workflow-related residual confounding while not excluding all possible unmeasured bias (Table 6). The procedural pseudo-score showed no association with TG z-score (ATE = +0.01, 95%CI: −0.06 to +0.08, *p* = 0.78), LDL-C z-score (ATE = -0.02, 95%CI: −0.09 to +0.05, *p* = 0.59), or CRP z-score (ATE = +0.02, 95%CI: −0.05 to +0.09, *p* = 0.61). Similarly, syndrome load exhibited no association with the negative control outcome of the incident documentation of a simple renal cortical cyst notation (OR = 1.03, 95%CI: 0.88–1.21, *p* = 0.72).

**Table 6:** Sensitivity analyses: negative controls, E-values, baseline-laboratory models, and weighting robustness.

Analysis	Outcome/Estimate	Point estimate/E-value	CI-limit E-value	Comment
Negative control exposure				
Procedural pseudo-score	TG z-score	+0.01 (−0.06 to +0.08)	–	Null association
Procedural pseudo-score	LDL-C z-score	−0.02 (−0.09 to +0.05)	–	Null association
Procedural pseudo-score	CRP z-score	+0.02 (−0.05 to +0.09)	–	Null association
Negative control outcome				
Syndrome load	Incident simple renal cortical cyst (OR)	1.03 (0.88–1.21)	–	Null association
E-values for primary effects				
Syndrome load— > TG z-score	+0.18 (0.12–0.24)	1.92	1.54	RR-equivalent from SMD
Syndrome load— > LDL-C z-score	+0.11 (0.05–0.18)	1.54	1.27	RR-equivalent from SMD
Syndrome load— > CRP z-score	+0.16 (0.09–0.23)	1.76	1.44	RR-equivalent from SMD
Baseline-laboratory sensitivity variants				
CRP-excluded nuisance specification	CRP z-score	+0.14 (+0.07 to +0.21)	–	Attenuated but directionally stable
Reduced laboratory GPS model	TG z-score	+0.16 (+0.10 to +0.23)	–	Stable direction
Reduced laboratory GPS model	LDL-C z-score	+0.09 (+0.03 to +0.16)	–	Stable direction
Reduced laboratory GPS model	CRP z-score	+0.13 (+0.06 to +0.20)	–	Attenuated but stable
Weight truncation variants				
0.5%/99.5% truncation	TG z-score	+0.17 (0.11–0.23)	–	Stable estimate
2%/98% truncation	TG z-score	+0.19 (0.13–0.25)	–	Stable estimate
CBPS alternative	TG z-score	+0.18 (0.11–0.25)	–	Stable estimate

TG, triglycerides; LDL-C, low-density lipoprotein cholesterol; CRP, C-reactive protein; OR, odds ratio; CI, confidence interval; CBPS, covariate balancing propensity score. Negative controls were prespecified to have no plausible short-term biological pathway to lipid or CRP outcomes and were used to probe gross workflow-related bias; null results do not prove absence of all unmeasured confounding. E-values for continuous outcomes were calculated after translating standardized mean differences to approximate risk-ratio equivalents. Baseline-laboratory sensitivity analyses evaluated whether primary estimates depended materially on baseline CRP or other time-zero laboratory severity markers.

E-values provided quantitative sensitivity parameters (Table 6). For the TG z-score association (+0.18), the approximate risk ratio equivalent yielded an E-value of 1.92, indicating that an unmeasured confounder would need associations of at least 1.92-fold with both syndrome load and TG to fully explain the observed association; the E-value for the confidence limit closest to the null was 1.54. Similar patterns emerged for LDL-C (point estimate E-value 1.54) and CRP (point estimate E-value 1.76).

Weight truncation sensitivity analyses yielded consistent results: Varying truncation thresholds (0.5%/99.5% vs. 1%/99% vs. 2%/98%) altered TG z-score ATE estimates minimally (+0.17 to +0.19). Weighted balance remained acceptable across exposure quintiles in all variants (maximum post-weighting absolute SMD 0.08). Substituting CBPS-style balancing weights for the primary GPS model produced nearly identical estimates (TG z-score ATE = +0.18, 95%CI: 0.11–0.25).

Baseline laboratory sensitivity analyses directly addressed the concern that baseline CRP and related time-zero laboratory severity markers might induce overconditioning. When baseline CRP was omitted from the CRP-specific GPS/DML nuisance specification, the CRP z-score association attenuated modestly from +0.16 to +0.14 (95%CI: +0.07 to +0.21). In the reduced laboratory model excluding baseline inflammatory/metabolic biomarkers from the propensity score, estimates remained directionally consistent for TG (+0.16; 95%CI: +0.10 to +0.23), LDL-C (+0.09; 95%CI: +0.03 to +0.16), and CRP (+0.13; 95%CI: +0.06 to +0.20). These sensitivity analyses did not change the qualitative conclusion but reinforced cautious interpretation of CRP as an adjusted intervention–analog association rather than a controlled direct effect.

## 4 Discussion

This single-center retrospective cohort study leveraged rigorous latent variable modeling and target-trial-inspired causal inference methodology to demonstrate reproducible, dose-dependent associations between TCM syndrome load and objective lipid and CRP biomarkers. By constructing a syndrome load index with established measurement invariance across sex and age groups, explicitly specifying an intervention–analog contrast, and applying continuous GPS weighting combined with DML, we provide evidence that higher syndrome load is associated with small-to-moderate worsening of dyslipidemia and systemic inflammation. These findings advance TCM syndrome objectification and suggest potential clinical utility for syndrome-based cardiometabolic risk stratification, while not proving that the latent syndrome construct itself is directly manipulable.

The observed magnitudes—approximately +0.10 mmol/L for LDL-C and +0.20 mmol/L for TG per 1-SD higher syndrome load—are modest at the individual level but clinically interpretable at the population level. The corresponding 4.6% absolute increase in hypertriglyceridemia risk implies roughly one additional abnormal TG result for every 22 patients with syndrome load 1 SD higher than otherwise similar comparators. In contemporary risk frameworks such as SCORE2, shifts of this magnitude would not by themselves redefine risk category, but in older adults, patients with diabetes, or those with obesity, they may contribute meaningfully to cumulative residual risk when added to conventional factors (24, 25). The stronger association observed with CRP than with LDL-C is also consistent with the growing recognition that residual inflammatory risk contributes to cardiovascular vulnerability (27–30).

The heterogeneous associations merit particular attention. Syndrome load associations were amplified among older adults, individuals with diabetes, and those with higher BMI—precisely the subgroups already prioritized for intensive prevention. This pattern suggests that syndrome load may enhance risk stratification beyond conventional models, potentially identifying patients who may benefit from intensified lifestyle counseling, closer biochemical monitoring, or prospectively tested syndrome-targeted treatment strategies. The persistence of associations among statin users implies that syndrome-related signals are not exhausted by LDL lowering alone, aligning with residual inflammatory risk concepts increasingly recognized in cardiovascular prevention (29, 30).

Several methodological strengths warrant emphasis. First, measurement invariance testing via CFA and IRT supported equivalent interpretation of syndrome scores across demographic groups and therefore strengthened descriptive comparability and subgroup interpretation; it was not treated as evidence for exchangeability or confounding control (31, 32). Second, explicit target trial specification clarified the estimand, made time zero transparent, and reduced risks of self-inflicted biases such as immortal time bias (13, 33). Third, combining continuous GPS-based IPTW with DML provided doubly robust estimates resilient to moderate model misspecification under the stated assumptions (18, 34). Fourth, negative control analyses, E-value calculations, and baseline laboratory sensitivity models offered transparent sensitivity assessments, enhancing confidence in cautious intervention–analog interpretations (35, 36). Finally, framing syndrome load as an intervention analog, rather than a literal randomized latent trait, narrows the interpretation to feasible syndrome-shifting strategies in routine care.

This single-center study has several limitations. First, syndrome load is a reflective latent exposure derived from tongue, pulse, and symptom indicators; it should not be interpreted as a directly manipulable treatment in the same sense as blood pressure, LDL-C, or a prescribed medication. The 1-SD contrast is therefore an intervention–analog scaling of the syndrome index, and prospective trials targeting concrete syndrome manifestations are required to test whether modifying these manifestations changes lipid or inflammatory biomarkers. Second, baseline syndrome load originated from subjective, non-blinded four-diagnostic TCM assessments; although CFA/IRT improved reliability and cross-group comparability, the latent exposure still inherits some measurement error and observer dependence. Third, index-visit and post-baseline treatment decisions could be influenced by syndrome severity and could subsequently affect 90-day biomarkers; these decisions were treated as co-interventions downstream of time zero rather than baseline confounders; therefore, the estimates should be interpreted as pragmatic intervention–analog associations rather than pure biological effects. Fourth, baseline laboratory variables, particularly CRP, may capture both pre-time-zero severity and early inflammatory expression of syndrome manifestations, creating possible overadjustment or collider concerns; reduced-adjustment sensitivity analyses produced attenuated but directionally consistent estimates, which support the robustness of direction but do not eliminate this interpretive limitation. Fifth, the study was geographically localized, and syndrome interpretation can vary across institutions, provinces, and TCM schools of practice, limiting direct generalizability to other diagnostic traditions. Sixth, residual confounding remains possible, particularly from incompletely measured diet timing, sleep disruption, shift work, physical activity, and other chronobiological factors that influence inflammation and lipid metabolism. Recent circadian-cardiometabolic literature, including a 2025 Sci Bull synthesis on intermittent fasting and cardiovascular health, suggests that timing-related exposures may modulate both inflammatory tone and lipid handling (37), and these factors should be prospectively captured in future multicenter studies. Seventh, the 90-day window supports short-term biomarker interpretation but may be too brief to characterize longer-term lipid remodeling or hard cardiovascular outcomes. Finally, as only CRP was available consistently in this retrospective dataset, the inflammatory domain was represented by a single biomarker rather than a broader cytokine panel. Future studies should assess whether syndrome load can be integrated into multimodal prediction systems and electronic health record pipelines, analogous to emerging AI-enabled cardiovascular prognostic frameworks such as recent deep-learning cardiac magnetic resonance models for STEMI risk prediction (38), while preserving the interpretability and clinical grounding of syndrome-based medicine.

## 5 Conclusion

TCM syndrome load, rigorously measured and cautiously evaluated using CFA/IRT, explicit TTE specification, continuous GPS weighting, DML, and multiple sensitivity analyses, showed reproducible dose–response associations with dyslipidemia and CRP elevation. Associations were particularly pronounced among high-risk subgroups, suggesting potential utility for personalized risk assessment and supporting prospective intervention studies that target concrete syndrome manifestations rather than the latent construct itself.

## Data Availability

The original contributions presented in the study are included in the article/Supplementary material, further inquiries can be directed to the corresponding author.

## References

[1] Liu JY Li X. Standardization, objectification, and essence research of traditional Chinese medicine syndrome: a 15-year bibliometric and content analysis from 2006 to 2020 in web of science database. Anat Rec (Hoboken). (2023) 306:2974–83. doi: 10.1002/ar.24821, 34739744

[2] Gong L Jiang J Chen S Qi M. A syndrome differentiation model of TCM based on multi-label deep forest using biomedical text mining. Front Genet. (2023) 14:1272016. doi: 10.3389/fgene.2023.1272016, 37854059 PMC10579813

[3] Li R Ma T Gu J Liang X Li S. Imbalanced network biomarkers for traditional Chinese medicine syndrome in gastritis patients. Sci Rep. (2013) 3:1543. doi: 10.1038/srep01543, 23529020 PMC3607832

[4] Zhang X Tian R Zhao C Birch S Lee JA Alraek T . The use of pattern differentiation in WHO-registered traditional Chinese medicine trials-a systematic review. Eur J Integr Med. (2019) 30:100945. doi: 10.1016/j.eujim.2019.100945

[5] Huang Z Miao J Chen J Zhong Y Yang S Ma Y . A traditional Chinese medicine syndrome classification model based on cross-feature generation by convolution neural network: model development and validation. JMIR Med Inform. (2022) 10:e29290. doi: 10.2196/29290, 35384854 PMC9021949

[6] Gan L Jiang TT Yi WJ Lu R Xu FY Liu CM . Study on potential biomarkers of energy metabolism-related to early-stage yin-deficiency-heat syndrome based on metabolomics and transcriptomics. Anat Rec (Hoboken). (2020) 303:2109–20. doi: 10.1002/ar.24355, 31909898

[7] Yue W Ji W Wang X Ma X Wang P Wang X. SDRP: Prescription recommendation with syndrome differentiation in traditional Chinese medicine. IEEE J Biomed Health Inform. (2025) 29:3736–49. doi: 10.1109/JBHI.2025.3525507 40031024

[8] Dai J Sun S Cao H Zheng N Wang W Gou X . Applications of new technologies and new methods in ZHENG differentiation. Evid Based Complement Alternat Med. (2012) 2012:298014. doi: 10.1155/2012/298014, 22675378 PMC3364574

[9] Liu J Liu P Dai R Liu Y Wang J Zheng Y . Analysis of plasma proteome from cases of the different traditional Chinese medicine syndromes in patients with chronic hepatitis B. J Pharm Biomed Anal. (2012) 59:173–8. doi: 10.1016/j.jpba.2011.10.002, 22030074

[10] Lo LC Chen CY Chiang JY Cheng TL Lin HJ Chang HH. Tongue diagnosis of traditional Chinese medicine for rheumatoid arthritis. Afr J Tradit Complement Altern Med. (2013) 10:360–9. doi: 10.4314/ajtcam.v10i5.24, 24311851 PMC3847431

[11] Jiang B Liang X Chen Y Ma T Liu L Li J . Integrating next-generation sequencing and traditional tongue diagnosis to determine tongue coating microbiome. Sci Rep. (2012) 2:936. doi: 10.1038/srep00936, 23226834 PMC3515809

[12] Hernán MA Robins JM. Using big data to emulate a target trial when a randomized trial is not available. Am J Epidemiol. (2016) 183:758–64. doi: 10.1093/aje/kwv254, 26994063 PMC4832051

[13] Fu EL. Target trial emulation to improve causal inference from observational data: what, why, and how? J Am Soc Nephrol. (2023) 34:1305–14. doi: 10.1681/ASN.0000000000000152, 37131279 PMC10400102

[14] Hernán MA Dahabreh IJ Dickerman BA Swanson SA. The target trial framework for causal inference from observational data: why and when is it helpful? Ann Intern Med. (2025) 178:402–7. doi: 10.7326/ANNALS-24-01871, 39961105 PMC11936718

[15] Hernán MA Wang W Leaf DE. Target trial emulation: a framework for causal inference from observational data. JAMA. (2022) 328:2446–7. doi: 10.1001/jama.2022.21383, 36508210

[16] Putnick DL Bornstein MH. Measurement invariance conventions and reporting: the state of the art and future directions for psychological research. Dev Rev. (2016) 41:71–90. doi: 10.1016/j.dr.2016.06.004, 27942093 PMC5145197

[17] Muthén B Asparouhov T. IRT studies of many groups: the alignment method. Front Psychol. (2014) 5:978. doi: 10.3389/fpsyg.2014.00978, 25309470 PMC4162377

[18] Chernozhukov V Chetverikov D Demirer M Duflo E Hansen C Newey W . Double/debiased machine learning for treatment and structural parameters. Econometrics J. (2018) 21:C1–C68. doi: 10.1111/ectj.12097

[19] Bach P Chernozhukov V Kurz MS Spindler M. DoubleML – an object-oriented implementation of double machine learning in Python. J Mach Learn Res. (2022) 23:2495–501. doi: 10.48550/arXiv.2104.03220

[20] Lipsitch M Tchetgen Tchetgen E Cohen T. Negative controls: a tool for detecting confounding and bias in observational studies. Epidemiology. (2010) 21:383–8. doi: 10.1097/EDE.0b013e3181d61eeb, 20335814 PMC3053408

[21] VanderWeele TJ Ding P. Sensitivity analysis in observational research: introducing the E-value. Ann Intern Med. (2017) 167:268–74. doi: 10.7326/M16-2607, 28693043

[22] Linden A Mathur MB VanderWeele TJ. Conducting sensitivity analysis for unmeasured confounding in observational studies using E-values: the evalue package. Stata J Promot Commun Stat Stata. (2020) 20:162–75. doi: 10.1177/1536867x20909696

[23] Grundy SM Stone NJ Bailey AL Beam C Birtcher KK Blumenthal RS . 2018 AHA/ACC/AACVPR/AAPA/ABC/ACPM/ADA/AGS/APhA/ASPC/NLA/PCNA guideline on the management of blood cholesterol: a report of the American College of Cardiology/American Heart Association task force on clinical practice guidelines. Circulation. (2019) 139:e1082–143. doi: 10.1161/CIR.0000000000000625, 30586774 PMC7403606

[24] van der Leeuw J Ridker PM van der Graaf Y Visseren FLJ. Personalized cardiovascular disease prevention by applying individualized prediction of treatment effects. Eur Heart J. (2014) 35:837–43. doi: 10.1093/eurheartj/ehu004, 24513790

[25] SCORE2 Working Group, ESC Cardiovascular Risk Collaboration. SCORE2 risk prediction algorithms: new models to estimate 10-year risk of cardiovascular disease in Europe. Eur Heart J. (2021) 42:2439–54. doi: 10.1093/eurheartj/ehab309, 34120177 PMC8248998

[26] Zhou X Chen S Liu B Zhang R Wang Y Li P . Development of traditional Chinese medicine clinical data warehouse for medical knowledge discovery and decision support. Artif Intell Med. (2010) 48:139–52. doi: 10.1016/j.artmed.2009.07.012, 20122820

[27] Ridker PM Rifai N Rose L Buring JE Cook NR. Comparison of C-reactive protein and low-density lipoprotein cholesterol levels in the prediction of first cardiovascular events. N Engl J Med. (2002) 347:1557–65. doi: 10.1056/NEJMoa021993, 12432042

[28] Ridker PM Buring JE Shih J Rifai N Lee IM Buring JE. Inflammation, cholesterol, lipoprotein(a), and 30-year cardiovascular outcomes in women. N Engl J Med. (2024) 391:1783–93. doi: 10.1056/NEJMoa2405182 PMC11711015 39216091

[29] Ridker PM Everett BM Pradhan A MacFadyen J Solomon DH Zaharris E . Low-dose methotrexate for the prevention of atherosclerotic events. N Engl J Med. (2019) 380:752–62. doi: 10.1056/NEJMoa1809798, 30415610 PMC6587584

[30] Ridker PM Lei L Louie MJ Haddad T Nicholls SJ Lincoff AM . Inflammation and cholesterol as predictors of cardiovascular events among 13,970 contemporary high-risk patients with statin intolerance. Circulation. (2024) 149:349–59. doi: 10.1161/CIRCULATIONAHA.123.066213 PMC10752259 37929602

[31] Meade AW Lautenschlager GJ. A comparison of item response theory and confirmatory factor analytic methodologies for establishing measurement equivalence/invariance. Organ Res Methods. (2004) 7:361–88. doi: 10.1177/1094428104268027

[32] Widaman KF Grimm KJ. "Advanced psychometrics: structural equation modeling and item response theory". In: Little TD, editor. The Oxford Handbook of Quantitative Methods, Vol. 2: Statistical Analysis. Oxford, United Kingdom: Oxford University Press (2014). p. 419–45.

[33] Hernán MA Sauer BC Hernández-Díaz S Platt R Shrier I. Specifying a target trial prevents immortal time bias and other self-inflicted injuries in observational analyses. J Clin Epidemiol. (2016) 79:70–5. doi: 10.1016/j.jclinepi.2016.04.014, 27237061 PMC5124536

[34] Austin PC Cafri G. Variance estimation when using inverse probability of treatment weighting (IPTW) with survival analysis. Stat Med. (2016) 35:5642–55. doi: 10.1002/sim.7084, 27549016 PMC5157758

[35] Ding P VanderWeele TJ. Sensitivity analysis without assumptions. Epidemiology. (2016) 27:368–77. doi: 10.1097/EDE.0000000000000457, 26841057 PMC4820664

[36] Greenland S. Accounting for uncertainty about investigator bias: disclosure is informative. J Epidemiol Community Health. (2009) 63:593–8. doi: 10.1136/jech.2008.084913, 19596837

[37] Zhang J Chen Y Zhong Y Wang Y Huang H Xu W . Intermittent fasting and cardiovascular health: a circadian rhythm-based approach. Sci Bull (Beijing). (2025) 70:2377–89. doi: 10.1016/j.scib.2025.05.017, 40480884

[38] Chen Y Jiang M Xia C Zhao H Ke P Chen S . A novel deep learning system for STEMI prognostic prediction from multi-sequence cardiac magnetic resonance. Sci Bull (Beijing). (2025) 70:4241–52. doi: 10.1016/j.scib.2025.11.027, 41314962

