# Supplementary material for: Intervention–analog association between traditional Chinese medicine syndrome load and lipid/C-reactive protein biomarkers: a single-center retrospective cohort study using target trial emulation
Source: Front Med (Lausanne). 2026 Jul 6;13:1789294. doi: 10.3389/fmed.2026.1789294 (PMC13382539; doi:10.3389/fmed.2026.1789294)
Supplement: Supplementary file 1 [file Supplementary_file_1.DOCX]

**Supplementary Figure S1. Covariate balance before and after generalized propensity score weighting. Absolute standardized mean differences were reduced to <0.10 for all prespecified confounders in the primary analysis.**


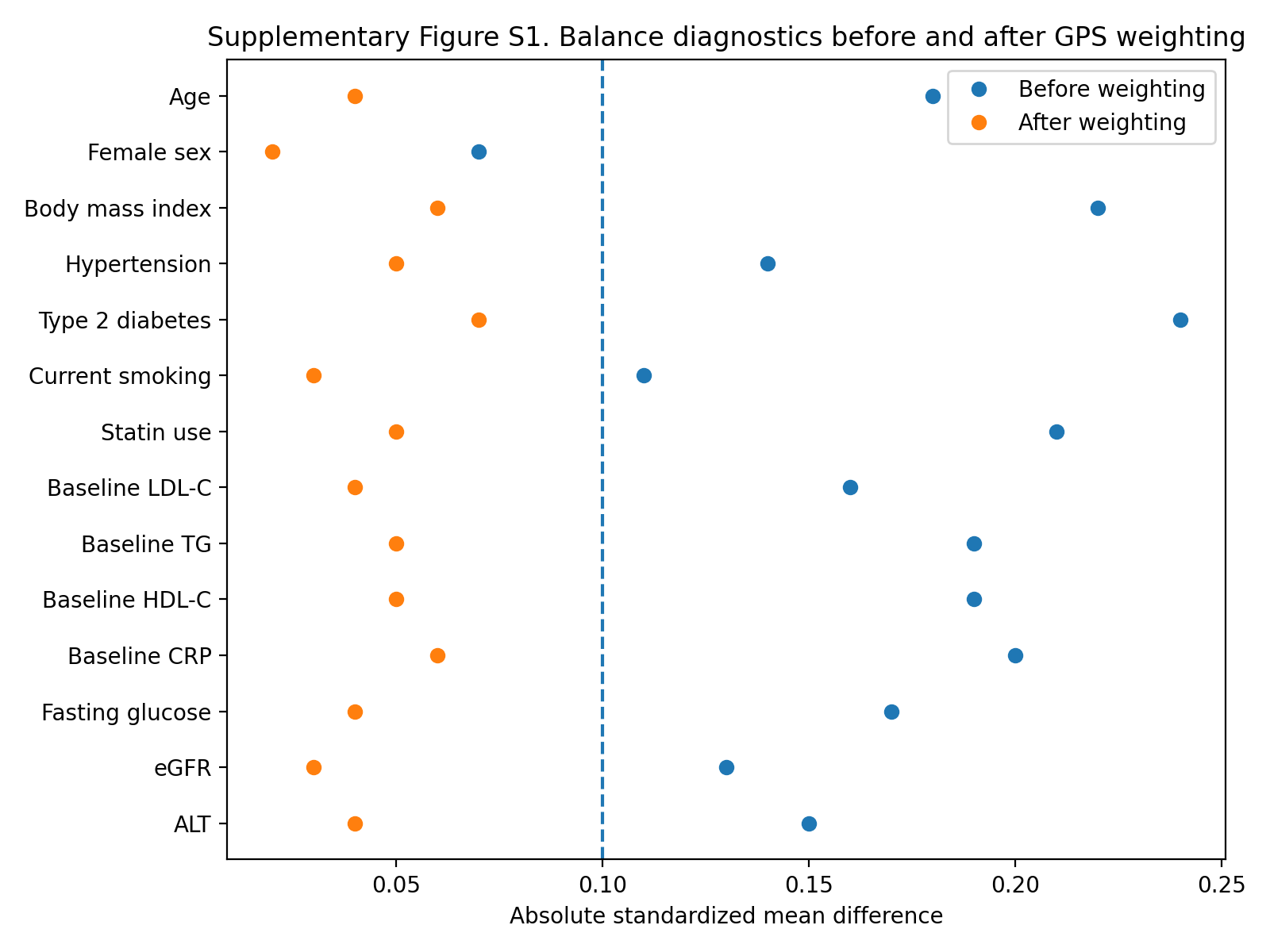


**Supplementary Figure S2. Distribution of stabilized generalized propensity weights. The distribution was centered near 1.0, consistent with good overlap and limited influence of extreme weights after truncation.**


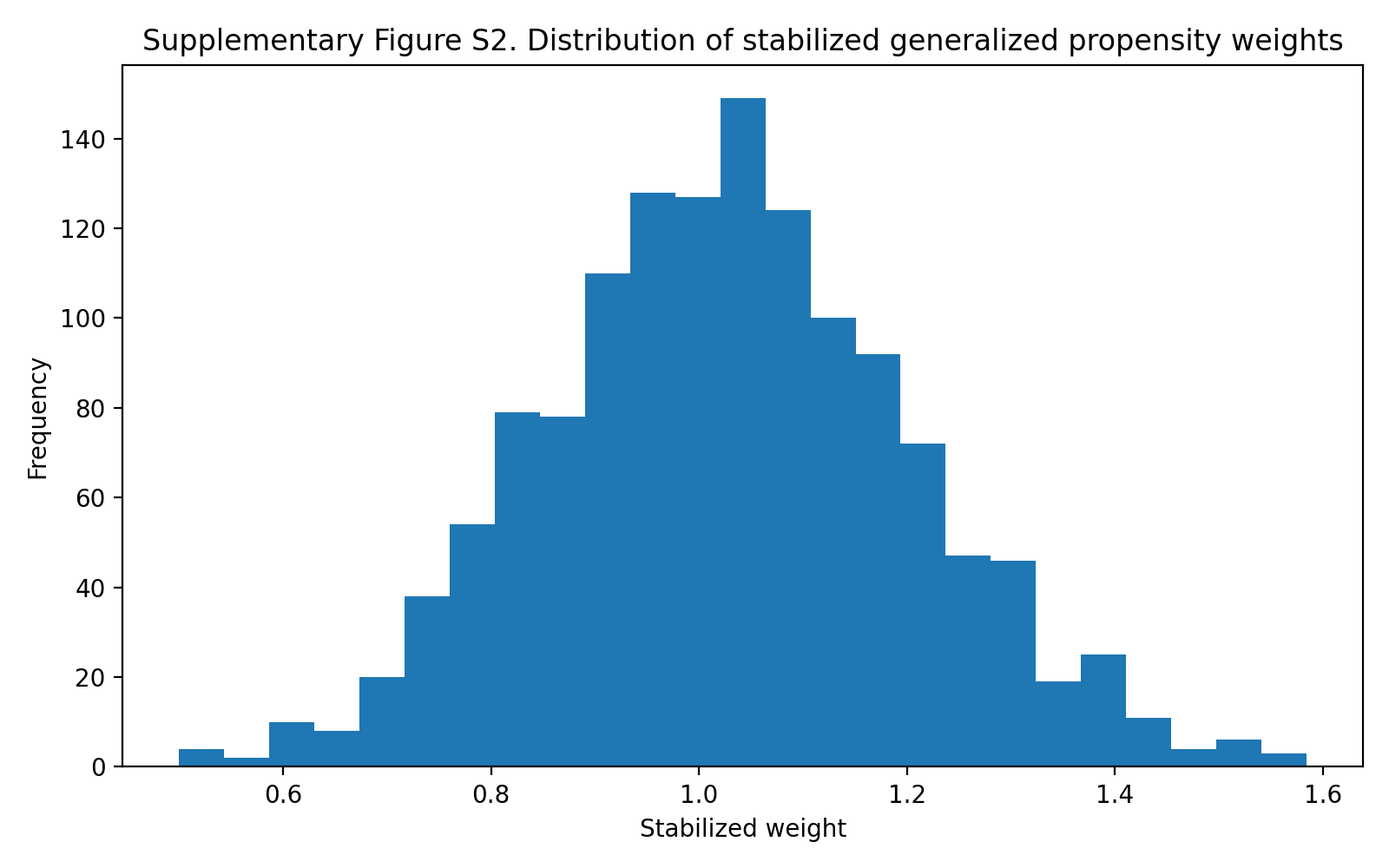


**Supplementary note: The primary exposure was modeled continuously using a Gaussian generalized propensity score. Quartiles of syndrome load were used only for descriptive visualization and overlap assessment.**

Supplementary note: Baseline-laboratory sensitivity analyses were added to evaluate whether primary estimates depended on baseline CRP or related time-zero laboratory severity markers. The CRP-specific estimate remained positive after omitting baseline CRP from nuisance models (+0.14, 95%CI +0.07 to +0.21), and a reduced laboratory GPS model yielded directionally consistent estimates for TG (+0.16), LDL-C (+0.09), and CRP (+0.13). These results support directional robustness while preserving cautious interpretation of CRP.
